# Phylodynamic reconstruction of H1N1pdm09 influenza virus transmission in Brazil: a decade of evolutionary dynamics

**DOI:** 10.1080/22221751.2026.2620237

**Published:** 2026-01-19

**Authors:** Isabela Carvalho Brcko, Vinicius Carius de Souza, Alex Ranieri Jeronimo Lima, James Siqueira Pereira, Evaldo Stanislau Affonso de Araújo, Ana Paula Nunes Viveiros Valeiros, Melissa Palmieri, Juliana Almeida Nunes, Leandro Spalato Torres, Hazerral de Oliveira Santos, Anderson Brandão Leite, Felicidade Mota Pereira, Arabela Leal e Silva de Mello, Vanessa Brandão Nardy, Gabriela Sant'Ana Menezes de Andrade, Marcela Kelly Astete Gomez, Lucas Luiz Vieira, Mariana Matos Roll, Brenno Vinícius Martins Henrique, Lídio Gonçalves Lima Neto, Elaine Cristina de Oliveira, Júlia Deffune Profeta Cidin Almeida, Stephanni Figueiredo da Silva, Klaucia Rodrigues Vasconcelos, Talita Emile Ribeiro Adelino, Natalia Rocha Guimaraes, Luiz Marcelo Ribeiro Tomé, Lavinia Nery Villa Stangler Arend, Ciciléia Correia da Silva, Adriana Cristina Salvador Maia, Cristiane Batista Mattos, Glaucilene da Silva Costa, Luiz Carlos Alcântara, Esper G. Kallás, Sandra Coccuzzo Sampaio, Svetoslav Nanev Slavov, Marta Giovanetti, Maria Carolina Elias

**Affiliations:** a Center for Viral Surveillance and Serological Assessment (CeVIVAS), Butantan Institute, São Paulo, Brazil; b Programa Interunidades de Pós-graduação em Bioinformática, Universidade de São Paulo, São Paulo, Brazil; c Santos Municipal Government, Santos, Brazil; d Faculdade de Medicina da Universidade São Judas Tadeu, Santos, Brazil; e Municipal Health Department, São Paulo Municipal Government, São Paulo, Brazil; f Central Public Health Laboratory of Alagoas (LACEN-AL), Maceió, Brazil; g Central Public Health Laboratory of Bahia (LACEN-BA), Salvador, Brazil; h Central Public Health Laboratory of Distrito Federal (LACEN-DF), Brasília, Brazil; i Central Public Health Laboratory of Maranhão (LACEN-MA), São Luis, Brazil; j Central Public Health Laboratory of Mato Grosso (LACEN-MT), Cuiabá, Brazil; k Ezequiel Dias Foundation (FUNED), Belo Horizonte, Brazil; l Central Public Health Laboratory of Paraná (LACEN-PR), Curitiba, Brazil; m Rene Rachou Institute, Oswaldo Cruz Foundation, Belo Horizonte, Brazil; n Butantan Institute, São Paulo, Brazil; o University of São Paulo Faculty of Medicine Clinics Hospital, São Paulo, Brazil; p Department of Science and Technology for Humans and the Environment, Università Campus Bio-Medico di Roma, Rome, Italy; q Climate Amplified Diseases And Epidemics (CLIMADE), Brazil, Americas

**Keywords:** H1N1pdm09, phylodynamics, whole-genome sequencing, Brazil, genomic surveillance

## Abstract

The H1N1pdm09 influenza virus, which emerged in 2009 following a unique reassortment of swine-origin gene segments, rapidly replaced the seasonal H1N1 strain and triggered the first influenza pandemic of the twenty-first century. In Brazil, the virus initially spread through intense community transmission before establishing a pattern of seasonal circulation. However, its long-term evolutionary dynamics in the country remain insufficiently characterized. To address this gap, we conducted a coordinated national genomic surveillance effort focused on the period from 2014 onward, when Brazil began systematic whole-genome sequencing of circulating H1N1pdm09 viruses. Through collaborative sequencing across all five Brazilian macroregions, we generated 597 complete genomes collected between 2014 and 2024. Using phylodynamic approaches, we reconstructed the spatiotemporal spread of H1N1pdm09, identified major circulating lineages, and integrated epidemiological data to assess patterns of persistence and regional transmission. Our findings reveal sustained circulation and multiple independent viral introductions over the past decade, with evidence of localized lineage maintenance, particularly in the Southeast and South regions. Phylogenetic analyses also indicate repeated seeding from international sources, underscoring the continued impact of global viral movement. In addition, genome-wide comparisons revealed reassortment events involving internal segments, which may have contributed to the persistence and adaptation of dominant lineages following the COVID-19 pandemic. This study presents the most comprehensive reconstruction of H1N1pdm09 evolutionary dynamics in Brazil to date, highlighting the critical role of integrated, nationwide genomic surveillance in enhancing public health preparedness in tropical and subtropical regions.

## Introduction

The emergence of the Influenza A/H1N1pdm09 virus in 2009 marked a pivotal moment in global influenza epidemiology. This novel strain originated through an unprecedented reassortment event, combining five gene segments from a North American triple reassortant swine lineage (PB2, PB1, PA, NP, and NS), the HA (H1) from a classical H1N1 swine lineage, and two segments (NA (N1) and MP) from a Eurasian avian-like swine influenza lineage [1–3]. H1N1pdm09 rapidly displaced the seasonal H1N1 virus that had circulated in humans since 1918 [4], triggering the first influenza pandemic of the twenty-first century [5,6]. Phylogenetic evidence points to Mexico as the site of emergence in early 2009 [7,8]. In Brazil, the virus spread rapidly, with sustained community transmission occurring within months [9] and the country reporting disproportionately high morbidity and mortality compared to other affected nations [10].

Following the pandemic phase, H1N1pdm09 transitioned into seasonal circulation alongside A/H3N2 and Influenza B viruses [11,12], frequently dominating influenza seasons in Brazil [13–16]. This epidemiological shift raises key questions about the virus's long-term evolutionary trajectory and population dynamics in a tropical setting marked by climatic diversity and heterogeneous immunity. Although global surveillance has documented the evolution of H1N1pdm09, substantial knowledge gaps persist regarding its genetic diversity and transmission patterns within Brazil, particularly following its establishment as a seasonal pathogen.

Previous studies of H1N1pdm09 in Brazil have largely focused on partial genomic data, most notably the hemagglutinin (HA) segment, to infer evolutionary patterns and transmission dynamics [12], or were restricted to specific regions [17]. More recently, lineage-level distinctions among circulating Influenza A strains in Brazil have been reported [18]. However, a comprehensive understanding of viral spread and adaptation requires full-genome analyses, which only became systematically available in Brazil from 2014 onwards. Whole-genome data are essential for identifying reassortment events, mapping fine-scale transmission routes, and detecting adaptive mutations that may influence viral fitness, transmissibility, and seasonal dominance.

Here, we present a nationwide genomic analysis of H1N1pdm09 in Brazil covering the period 2014–2024, which marks the beginning of systematic whole-genome sequencing in the country and captures the virus’s post-pandemic evolutionary trajectory. As part of a coordinated national surveillance effort, led by the Center for Viral Surveillance and Serological Assessment (CeVIVAS) of Butantan Institute (https://cevivas.butantan.gov.br/, accessed July 2025), we generated 597 complete H1N1pdm09 genomes from all five Brazilian macroregions, enabling a comprehensive assessment of the virus’s long-term evolutionary dynamics. By integrating genomic, epidemiological, and phylodynamic analysis, we reconstructed the spatiotemporal spread of H1N1pdm09, identified major circulating lineages and their global connections, and explored patterns of persistence, diversification, and reintroduction. This study fills critical gaps in our understanding of H1N1pdm09 evolution in Brazil and underscores the importance of unified genomic surveillance strategies to inform influenza monitoring, preparedness, and response in tropical and subtropical regions.

## Materials and methods

### Ethical considerations

The study protocol was reviewed and approved by the Institutional Ethics Committee of the Faculty of Medicine, University of São Paulo (CAAE: 68586623.0.0000.0068). Samples generated in this study were obtained as anonymous clinical specimens from material exceeding routine algorithms for Respiratory Syndrome diagnostics performed in public health laboratories within the Brazilian Ministry of Health (MS) network.

### Molecular screening and whole genome sequencing of H1N1pdm09 Influenza A virus

Viral detection was performed using commercial qPCR kits, including GeneFinder™ COVID-19/FluA&B RealAmp and INFA/INFB/SC2-Bio-Manguinhos. Whole-genome sequencing was conducted for samples with cycle threshold (Ct) values below 30 and available epidemiological metadata, including collection date and location. Detailed protocols for purification, RNA extraction, sequencing, and genome assembly were described in 2025 by Brcko et al. [18]. After genome assembly, subtyping and clade attribution were performed by comparing the HA segment of each sample against the reference sequence H1-A/Wisconsin/588/2019 (MW626062) using Nextclade [19]. We generated a total number of 597 H1N1pdm09 whole-genome sequences; obtained from all five Brazilian macroregions: North (n = 7), Northeast (n = 244), Midwest (n = 65), Southeast (n = 241), and South (n = 40). Complete information regarding the genome sequences included in this study is provided in Table S1.

### Construction of a global reference dataset for phylogenetic analysis of H1N1pdm09

To contextualize the recent Brazilian H1N1pdm09 sequences within the broader global evolutionary history of the virus, we retrieved historical whole-genome sequences from the GISAID EpiFlu Database [20] (https://www.gisaid.org/, accessed December 2024). The dataset included complete genomes collected between 2014 and 2024 from Brazil and other countries, with human as the host, as whole-genome sequences from Brazil before 2014 were not available. We selected this time frame for analysis to ensure consistency and comparability. To minimize sampling bias in global comparisons, we downsampled non-Brazilian sequences using the augur filter tool from the Nextstrain pipeline [21], selecting up to three sequences per country, year, and month, and stratifying the dataset by continental regions to achieve equitable representation. This process yielded a representative dataset comprising 4,562 global and 1,622 national sequences. Upon integrating the CeVIVAS and GISAID datasets, we obtained a consolidated collection of 2,219 Brazilian sequences. All downstream analyses were conducted using the combined dataset of 6,781 sequences. Detailed information is provided in Tables S2 and S3 and Figure S1a and b.

### Phylogenetic and phylodynamic analysis of H1N1pdm09 Influenza A virus

Sequence alignments were performed using ViralMSA v1.1.44 [22] with Minimap2 [23], referencing the following segments: PB2 (MG027913.1), PB1 (CY121686.1), PA (MG027912.1), HA (NC_026433.1), NP (MG027915.1), NA (CY121682.1), MP (CY121681.1), and NS (CY121684.1). Alignments were manually inspected and trimmed using AliView [24]. Maximum likelihood (ML) phylogenetic trees were independently reconstructed for each segment using IQ-TREE2 [25], applying a general time reversible (GTR) nucleotide substitution model with a proportion of invariable sites. The temporal signal of each dataset was then assessed through root-to-tip regression in TempEst [26], and sequences with poor temporal fit were excluded from downstream analyses. ML trees were subsequently converted into time-scaled phylogenies using TreeTime [27]. Outlier sequences that deviated from the strict molecular clock assumption, as flagged by TreeTime, were removed with the Ape package in R [28] until a robust time-scaled phylogeny was obtained. For each viral population, time-scaled tree topologies were generated, and discrete ancestral state reconstruction of geographic locations was performed using the *mugration* extension of TreeTime under a GTR model. The *mugration* package systematically explores internal nodes and tips, to identify and infer potential transmission events across locations. Finally, to quantify viral movement events, we used a custom Python script to iterate through each annotated phylogeny from root to tip. A state change was recorded whenever an internal node transitioned from one location to a different location at the descendant internal node or tip. The timing of each transition was also documented, providing estimates of importation and exportation events. Tree visualization and data manipulation of phylogenetic analyses were conducted in R (v4.4.1; R Core Team, 2023) using in-house scripts for ggtree [29], ape [28], phytools [30], treeio [31], and ggplot2 [32].

To jointly infer evolutionary relationships and dispersal history for each viral segment, we applied a time-scaled Bayesian approach using BEAST v1.10.4 [33]. Analyses were conducted under the SRD06 partitioned substitution model, with a GTR nucleotide substitution model, an uncorrelated lognormal relaxed clock, and a Bayesian skyline coalescent prior [34]. Independent MCMC chains were run for 200 million iterations, sampling every 20,000 steps until all parameters achieved effective sample sizes (ESS) > 200. Chains were assessed for convergence using Tracer v1.7.1 [35]. After removing 10% burn-in, runs were combined with LogCombiner v1.10.4 and downsampled to a final posterior distribution of 2,000 trees. Maximum clade credibility (MCC) trees were summarized using TreeAnnotator v1.10.4, and 95% highest posterior density (HPD) intervals were used to express statistical uncertainty in parameter estimates.

### Selective pressure and recombination analysis

To investigate the selective pressures acting on the Brazilian A/H1N1pdm09 virus during the 2014–2024 period, we analyzed individual datasets for each of the nine viral proteins (PB2, PB1, PA, HA, NP, NA, M1, M2, and NS) using two complementary temporal approaches, both conducted at the population level. First, we estimated global dN/dS ratios over the entire study period using the Single-Likelihood Ancestor Counting (SLAC) and Fast Unconstrained Bayesian AppRoximation (FUBAR) methods implemented in HyPhy v.2.0.0 [36]. Second, to capture temporal trends in selective pressure [37], annual dN/dS estimates were also computed using SLAC.

To investigate segment-specific topological incongruence and potential reassortment in A/H1N1pdm09, we independently reconstructed maximum-likelihood (ML) phylogenies for PB2, PB1, PA, HA, NP, NA, MP, and NS, restricting analyses to samples with complete segment coverage to ensure identical tip sets across trees. Each tree was rooted using the oldest available genome and aligned by tip labels. Topological discordance was quantified using normalized Robinson–Foulds (RF) distances [38] computed with phangorn v.2.12.1 [39], while dendextend v1.19.0 [40] was used for dendrogram manipulation and comparative visualization. Given the central evolutionary role of HA, we constructed tanglegrams between HA and each of the other seven segments, connecting corresponding taxa using colour-coded lineages inferred from ML ancestral state reconstruction. Genome-wide incongruence was further summarized using a pairwise RF distance matrix, ranging from 0 (identical) to 1 (maximally divergent) [38].

## Results

### Epidemiological and genomic profile of H1N1pdm09 Influenza A generated in this study

**Figure 1.**
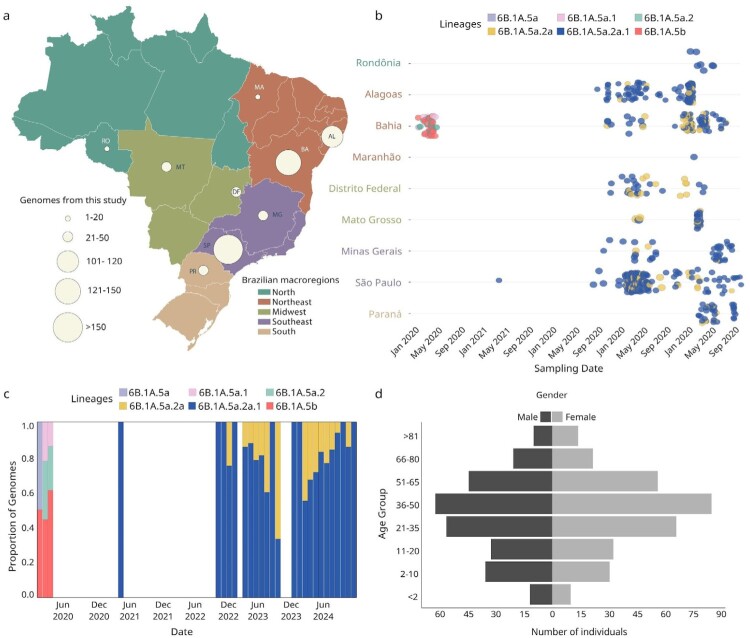
Genomic epidemiology of H1N1pdm09 influenza A virus in Brazil (2020–2024). (a) Map showing the number of H1N1pdm09 genome sequences generated per state, coloured by region and sized by genome count; (b) Timeline of H1N1pdm09 lineages identified in selected Brazilian states, with colours indicating distinct genetic clades; (c) Monthly lineage distribution of H1N1pdm09 genomes over time (2020–2024), expressed as the proportion of total sequences; (d) Distribution of sequenced cases by age group and gender.

A total of 597 complete genome sequences of Influenza A/H1N1pdm09 were generated from RT-qPCR-positive samples collected across Brazil between January 2020 and November 2024. The sequences were obtained from nine states: São Paulo (n = 196), Bahia (n = 142), Alagoas (n = 101), Minas Gerais (n = 45), Paraná (n = 40), Distrito Federal (n = 34), Mato Grosso (n = 31), Rondônia (n = 7), and Maranhão (n = 1), covering all five geographic macroregions of the country. The majority of genomes originate from the Northeast (n = 244) and Southeast (n = 241), together accounting for 81.2% of the total dataset (Figure 1(a) and Table S1).

Genomic characterization revealed the co-circulation of six distinct A/H1N1pdm09 lineages during the study period. In early 2020, sequences obtained predominantly from the Northeast region – where sampling was most concentrated – showed notable lineage diversity, with simultaneous detection of clades 6B.1A.5a, 6B.1A.5a.1, 6B.1A.5a.2, and 6B.1A.5b. A notable decline in genome recovery occurred between mid-2020 and mid-2022, coinciding with the peak of the COVID-19 pandemic and likely reflecting a temporary disruption in influenza surveillance efforts (Figure. 1(b, c)). From late 2022 onwards, a marked shift in the viral population structure was observed, with the emergence and eventual predominance of lineage 6B.1A.5a.2a and its descendant 6B.1A.5a.2a.1 across all sampled regions (Figure. 1(b, c)).

Demographic analysis of sequenced cases indicated a slight female predominance (n = 320) over males (n = 277). The median age of infected individuals was 37.4 years (range: 0–105 years) (Table S4), with infections reported across all age groups. The distribution of cases was skewed toward adults between 21 and 65 years of age, particularly those aged 36–50 years (25.0%), followed by 21–35 years (20.8%) and 51–65 years (17.1%). While overall sex distribution was balanced, minor differences were observed in the older age groups (Figure. 1(d)).

### Phylogenetic analysis and evolutionary dynamics of H1N1pdm09

**Figure 2.**
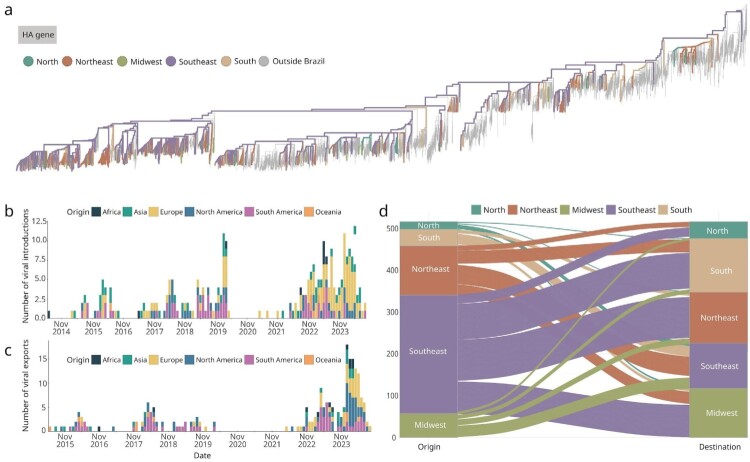
Phylogenetic and Evolutionary Dynamics of the HA gene of Influenza A/H1N1pdm09 in Brazil (2014–2024). (a) Time-resolved maximum-likelihood phylogeny of HA (hemagglutinin) gene, including high-quality genomic sequences from Brazil (n = 597) generated in this study, analyzed alongside other Brazilian and global reference sequences (n = 6,184); (b) Number of viral introductions into Brazil, representing external entries from international sources; (c) Number of viral exportations from Brazil to other regions of the world, representing internal sources of international spread; (d) Number of intra-national viral exchanges between Brazilian macroregions, estimated by tracking state changes from the root to the tips of the phylogeny shown in panel a.

**Figure 3.**
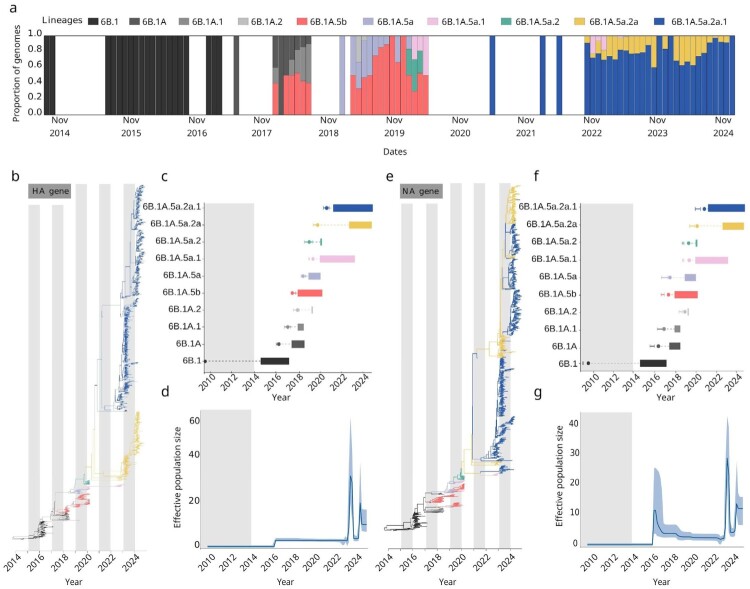
Temporal phylogenies and population dynamics of Influenza A/H1N1pdm09 virus in Brazil (2014–2024). (a) Proportion of circulating A/H1N1pdm09 lineages in Brazil from 2014 to 2024; (b, e) Maximum clade credibility (MCC) phylogenies of the HA (hemagglutinin) and NA (neuraminidase) genes, respectively, inferred using an uncorrelated lognormal relaxed clock model. The analyses included Brazilian sequences (n = 597, this study) and national reference sequences (n = 1,622). Branches are coloured by lineage. (c, f) Temporal persistence of lineages, showing a pattern of variant replacements over time. The x-axis represents the period from 2009 to 2024, while the y-axis shows the identified clades. Each clade is depicted with coloured bars indicating the span of its detection, with markers denoting the median time to the most recent common ancestor (tMRCA). Grey shading (2009–2014) denotes the interval without in-house sequence generation. (d, g) Population size dynamics for the HA and NA genes, respectively, over time were estimated using a Bayesian Skyline plot, with the effective population size on the y-axis and the time on the x-axis. The solid blue line represents the mean estimate, while the blue shaded area reflects the 95% highest posterior density (HPD) interval. Grey shading (2009–2014) denotes the interval without in-house sequence generation.

To investigate the evolutionary dynamics of A/H1N1pdm09 viruses following their establishment as seasonal influenza in Brazil, we integrated epidemiological data with phylodynamic analyses using a globally representative dataset of 4,562 genomes, including 2,219 sequences sampled in Brazil, between 6 January 2014 and 27 November 2024 (Figure 2(a) and Figure 3(a)).

Time-resolved phylogenetic reconstruction revealed the persistent co-circulation of multiple A/H1N1pdm lineages in Brazil, often interspersed with introductions from diverse international origins (Figure 2(a, b) and Figure 3(a, b)). These findings support a pattern of repeated viral importation events followed by local transmission and regional dissemination (Figure 2(a, b)). To ensure robustness, analyses were conducted on both the HA and NA gene segments – the two principal antigenic components of influenza A viruses.

Ancestral state reconstruction revealed the persistent co-circulation of multiple A/H1N1pdm09 lineages in Brazil, often interspersed with introductions from diverse international origins (Figure 2(b–d) and Figures S2b–d). These findings support a pattern of repeated viral importation events followed by local transmission and regional dissemination (Figure 2(b) and Figure S2b). To ensure robustness, analyses were conducted on both the hemagglutinin (HA) and neuraminidase (NA) segments – the two principal antigenic components of influenza A viruses. Ancestral state reconstruction applied to both gene trees enabled the identification of international viral importation and exportation events involving Brazil (Figure 2(b, c) and Figures S2b-c). Furthermore, this approach revealed intra-national viral exchanges among the five Brazilian macroregions: North, Northeast, Midwest, Southeast, and South (Figure 2(d) and Figure S2d).

In total, approximately 270 independent viral introductions into Brazil were inferred, most originating from Europe (Figure 2(b) and Figure S2b). Results were consistent across both the HA and NA datasets, reinforcing the overall conclusions. Nonetheless, minor segment-specific differences in tree topology and migration patterns suggest possible reassortment or recombination events – phenomena commonly observed in influenza virus evolution. A pronounced decline in international transmission events was detected following the implementation of COVID-19-related travel restrictions in April 2020, particularly affecting viral exportations from Brazil. From 2022 onwards, both international introductions and exportations gradually returned to pre-pandemic levels (Figure 2(b, c)and Figures S2b–c).

Analysis of intra-national viral spread identified the Southeast region as the primary hub of interregional transmission, accounting for 54.8% of inferred dissemination, followed by the Northeast with approximately 23% (Figure 2(d) and Figure S2d). Despite the lower sequence representation of the South region, it emerged as one of the main recipients of viral introductions – particularly from Southeast Brazil – highlighting its potential role as a domestic corridor for viral spread (Figure 2(d), and Figure S2d).

### Population dynamics and evolutionary turnover of A/H1N1pdm09 virus in Brazil (2014–2024)

To characterize the evolutionary patterns and population dynamics of A/H1N1pdm09 in Brazil, we analyzed time-scaled phylogenies and coalescent-based demographic reconstructions for both the HA and the NA genes (Figure 3). This dataset included 597 Brazilian sequences generated in this study and 1,622 national reference genomes, covering the period from 2014 to 2024. The proportional distribution of lineages (Figure 3(a)) revealed a clear succession of dominant clades over time. Clade 6B.1 predominated from 2014 to 2016, reflecting early post-pandemic viral establishment under conditions of limited diversity. From 2016 onward, the emergence of 6B.1A and its descendants marked a notable diversification phase. Between 2017 and 2018, multiple 6B.1A subclades co-circulated, with 6B.1A.5a rapidly becoming dominant. This lineage subsequently gave rise to 6B.1A.5a.2 and 6B.1A.5a.2a.1, which became the most prevalent variants in later years and persisted through 2024.

Time-scaled phylogenies of both HA and NA segments (Figure 3(b, e)) revealed concordant lineage replacement dynamics, suggesting parallel evolutionary trajectories at both major antigenic sites. Minor topological differences between segments, however, suggest occasional reassortment events, an expected feature of influenza virus evolution. Temporal persistence analyses (Figure 3(c, f)) confirmed ongoing lineage turnover, with replacement intervals typically ranging from one to three and a half years. Notably, 6B.1A.5a.2a and 6B.1A.5a.2a.1 displayed the longest persistence following their emergence post-2021. Median estimates of the time to the most recent common ancestor (tMRCA) clustered tightly among dominant lineages, consistent with rapid selective sweeps.

Bayesian Skyline plots (Figure 3(d, g)) revealed two major peaks in the effective population size. The first, in 2016, likely reflects the intense circulation of clade 6B.1 and the introduction of 6B.1A into Brazil, whereas the second, in 2023, is associated with the widespread expansion of clade 6B.1A.5a.2a and its descendant 6B.1A.5a.2a.1.

These demographic peaks suggest episodic increases in genetic diversity associated with the emergence of successful lineages. The demographic trajectories of HA and NA segments were largely congruent, although some segment-specific distinctions were observed. For instance, the NA segment displayed an earlier and sharper rise of the 6B.1A.5a lineage, while the HA segment exhibited a more gradual increase, suggesting the action of differential selective pressures on surface proteins.

Importantly, the impact of the COVID-19 pandemic was evident in both the evolutionary and demographic patterns. During 2020–2021, the effective population size of both HA and NA segments remained static at low levels (Figure 3(d, g)), likely reflecting reduced influenza transmission resulting from widespread non-pharmaceutical interventions, changes in healthcare-seeking behaviour, and disruptions to routine viral surveillance. This period coincided with a decline in lineage diversity and a temporary interruption of typical turnover patterns. Following the relaxation of containment measures in 2022, a rebound in diversity and viral population size was observed, culminating in the widespread circulation of 6B.1A.5a.2a.1 – likely facilitated by increased population susceptibility.

Further analysis of internal gene segments (Figure S3) supported and extended these findings. Time-scaled phylogenies of PB2, PB1, PA, NP, MP, and NS genes exhibited lineage structures largely consistent with those observed in HA and NA, reflecting genomic coherence across segments. Coalescent analyses of internal segments also revealed parallel trends, with gradual increases in effective population size between 2016 and 2019, followed by a sharp contraction during the pandemic and subsequent recovery post-2022. However, subtle segment-specific differences were noted. For instance, the PA segment exhibited a delayed first peak in 2018, while NP and MP showed more pronounced oscillations in population size. These variations may reflect segment-specific selective forces or differences in evolutionary rates.

### Selective pressure and events of recombination in A/H1N1pdm09 virus

Selective pressure analysis across all gene segments from 2015 to 2024 revealed consistently low dN/dS ratios in polymerase genes (PB2, PB1, PA), as well as in NP and M1, indicative of strong purifying selection. By contrast, the NS and M2 segments showed higher and more variable dN/dS values, with a coincident peak in 2020, suggesting episodic adaptive evolution or relaxed functional constraints. Overall, HA and NA exhibited intermediate dN/dS values but with distinct temporal patterns. From 2015 to 2019, HA increased subtly (≈0.1–0.3), whereas NA fluctuated more widely, with a peak in 2017 (≈0.5). After 2019, both genes stabilized around 0.2–0.3 but tended to vary inversely, a pattern that persisted through 2024 (Figure S4), suggesting compensatory selective dynamics that help maintain functional balance between the two surface proteins.

Complementing these gene-wide dN/dS patterns, site-level analyses (SLAC and FUBAR) identified discrete positively selected codons concentrated in antigenic or functionally relevant regions (Table S6). In HA, six codons exceeded neutrality thresholds (6, 11, 62, 86, 158, 233), several mapping to known antigenic sites. NA harboured two positively selected positions (77, 321), with FUBAR confirming site 77 in the stalk region. In M2, the positively selected sites, 21 and 23, correspond to known mutations conferring amantadine resistance. Additional positively selected codons were detected in NP (186, 217, 313), PB1 (384, 460), PB2 (184), and NS1 (84, 178, 205), though global dN/dS values in these segments remained low, indicating that adaptive substitutions occur against a background of predominant purifying selection.

To evaluate whether these segment-specific selective pressures were mirrored in phylogenetic structure, we compared maximum-likelihood trees for all eight segments using HA as a reference (Figure S5). Normalized Robinson–Foulds (RF) distances revealed substantial topological discordance between HA and the polymerase and NA trees (PB2 RF = 0.85, PB1 RF = 0.86, PA RF = 0.86, NA RF = 0.87), indicating heterogeneous evolutionary dynamics across the genome rather than fully coordinated trajectories. This pattern was consistent with the genome-wide RF distance matrix (Figure S6), which showed similarly high discordance among multiple segment pairs, reinforcing that incongruence is not restricted to HA-centric comparisons but reflects broader segment-level heterogeneity.

The tanglegram comparisons showed that topological incongruence was not uniformly distributed across the phylogeny. Clades corresponding to lineages 6B.1A.5a.2a and 6B.1A.5a.2a.1 exhibited consistently lower similarity between HA and multiple internal-gene topologies, with visible cross-tree displacements across several segments. These multi-segment mismatches are compatible with potential reassortment but do not constitute quantitative estimates of reassortment intensity. A comparable pattern of reduced similarity was also observed for lineage 6B.1A.5b in the HA–NS comparison.

## Discussion

Since its emergence in 2009, the A/H1N1pdm09 virus has become a recurrent cause of seasonal influenza in Brazil, characterized by heterogeneous transmission patterns, regional outbreaks, and a notable capacity for genetic diversification. In a country with continental dimensions and disparate health infrastructure, understanding the virus’s evolutionary and epidemiological dynamics is essential for optimizing surveillance strategies and guiding vaccine formulation.

In this study, we present a decade-long phylodynamic overview of A/H1N1pdm09 circulation in Brazil, integrating genomic, spatial, and demographic data to capture the complexity of viral evolution and transmission. Our findings reveal that, even in the absence of large-scale outbreaks, A/H1N1pdm09 has maintained endemic circulation, driven by fluctuating regional dynamics, the co-circulation of multiple lineages, and recurrent viral introductions. These patterns are consistent with global observations of persistent genetic diversification, often occurring below the detection threshold of traditional surveillance systems [41,42].

Demographic analysis of sequenced cases indicates a disproportionate burden of infection among working-age adults, particularly individuals aged 36–50 years. This finding aligns with previous studies from Brazil [17] and is likely influenced by occupational exposure and patterns of urban mobility [43,44]. These observations underscore the need to broaden vaccine prioritization strategies beyond age-based criteria to be included in current national frameworks.

Spatial analyses identified the Southeast and Southern regions as major hubs of viral dissemination, echoing earlier findings for both Influenza [45] and SARS-CoV-2 [46]. Factors such as population density (the largest Brazilian cities and international airports) and transportation infrastructure likely amplify these regions’ roles as hotspots for viral spread, emphasizing the need to prioritize them in national surveillance planning [47]. Additional transmission corridors, particularly Southeast – Northeast and South – Midwest, extend previous insights by Almeida et al. [48], suggesting that national viral spread is not limited to major urban centres, but may also follow alternative, often underappreciated, mobility routes. These insights are critical for refining national outbreak response strategies.

The concurrent circulation of multiple A/H1N1pdm09 lineages without full replacement reflects a complex interplay of partial cross-immunity, regional vaccine coverage disparities, and antigenic drift. Amino acid divergence between Brazilian strains and vaccine components [18] highlights ongoing immune escape. Although vaccination is often linked to increased viral diversification, data from SARS-CoV-2 suggest it may instead limit within-host evolution through stronger purifying selection [49]. Epidemic models further show that weak cross-immunity combined with host isolation can enable less fit viral strains to persist even under low reproduction numbers. Additionally, individual immunological history, shaped by prior infections or vaccination, modulates susceptibility and infectiousness, influencing viral coexistence [50].

In this context, although antigenic drift contributed to the low-level circulation of A/H1N1pdm09 during the pandemic years, its post-2022 resurgence, particularly of lineages 6B.1A.5a.2a and 6B.1A.5a.2a.1, aligns with the emerging “immunity debt” hypothesis. This hypothesis, still under investigation, posits that prolonged reductions in viral exposure due to stringent public health measures created a temporary gap in population immunity, thereby increasing susceptibility and amplifying the post-pandemic influenza burden [51]. Recent epidemiological analyses from Brazil indicate that the pandemic disrupted both the timing and magnitude of influenza activity [52], and our genomic analyses demonstrate that this epidemiological perturbation was paralleled by genomic and spatial restructuring of A/H1N1pdm09, including the expansion of lineages 6B.1A.5a.2a and 6B.1A.5a.2a.1.

Clade-frequency patterns further support this interpretation: while global datasets exhibited a largely synchronous post-2021 rise of 6B.1A.5a derivatives (Figures S7a–b), Brazil showed an earlier establishment of 6B.1A.5a.2a.1 and prolonged persistence of older 6B.1A.5a variants (Figure S7c). These regional deviations suggest that local epidemiological conditions and heterogeneous surveillance intensity modulated the tempo of lineage turnover in Brazil relative to other countries.

The marked rise in effective population size in 2023 (Figures S1 and S2) is consistent with this rebound, and the dominance of A/H3N2 in 2022 [18] may have further delayed the reestablishment of A/H1N1pdm09 transmission. Importantly, recent evidence suggests that the decline in antibody-mediated protection was driven more by antigenic drift than by waning immunity [53]. This finding raises particular concern for children, who may have missed critical windows of immune priming due to reduced viral circulation, potentially leaving them more vulnerable to infection. Together, these findings highlight the need to reinforce pediatric vaccination strategies and emphasize the relevance of updated genomic surveillance data for informing national and global vaccine-strain selection.

Beyond host-level susceptibility, the expansion of these resurgent lineages may also reflect intrinsic viral adaptations. Maximum likelihood tanglegrams revealed topological incongruences between the HA gene and internal segments (NP, MP, and NS), indicating reassortment events enriched in recently circulating lineages (Figure S3). These events, key drivers of influenza virus evolution [54,55] were non-randomly distributed across the phylogeny and appeared more frequently in persistent clades such as 6B.1A.5a.2 and 6B.1A.5a.2a.1. Such reassorted gene constellations may enhance viral fitness under immune pressure, with functionally compatible combinations of HA and internal segments potentially improving replication efficiency, immune evasion, or genome packaging [56]. The original emergence of A/H1N1pdm09 in 2009, itself the result of complex reassortment involving internal swine-origin [1,6] underscores the evolutionary significance of non-antigenic genes. While antigenic drift in HA remains central to vaccine strain selection, these findings highlight the added value of whole-genome sequencing for uncovering reassortment and segment-specific dynamics that would be missed in HA-only surveillance approaches.

Selective pressure analysis of A/H1N1pdm09 in Brazil reveals a predominantly purifying evolutionary pattern. Core segments, including the polymerase complex (PB2, PB1, PA), nucleoprotein (NP), and matrix protein 1 (M1), consistently exhibited strong purifying selection (dN/dS ≤ 0.21), reflecting their essential roles in replication and structure. Notably, the NS and M2 segments displayed occasional elevations in dN/dS values, reaching ∼0.49 for NS in 2017 and 2020, and peaking at 0.86 for M2 in 2020, before returning to baseline. These fluctuations coincided with reassortment events involving the NS and MP segments, suggesting possible transient shifts in selective pressure linked to genomic reorganization [57]. Surface antigens HA and NA exhibited modest increases in dN/dS during 2022–2023 (∼0.30 and ∼0.27, respectively), likely reflecting renewed antigenic evolution following COVID-19-related transmission bottlenecks. The absence of genomic data for 2021 underscores the importance of uninterrupted genome-wide surveillance to capture both gradual and episodic evolutionary changes.

More than virological insights, our findings speak to broader systemic challenges in infectious disease surveillance in emerging economies. Regional disparities in sequencing capacity, often shaped by the scope and timing of institutional collaborations, underscore the need to enhance the equity and representativeness of genomic monitoring. Since 2020, Brazil has made important strides in decentralizing its surveillance network through the Ministry of Health initiatives that expanded national whole-genome sequencing capabilities [58]. The establishment of the CeVIVAS initiative further extended these efforts to influenza, improving national genomic surveillance coverage. Although sequences were obtained from all five Brazilian macroregions, some, such as the North, were comparatively less represented. This sampling disparity may have influenced the inferred regional roles in the transmission network but likely reflects structural, financial, and logistical challenges inherent to conducting nationwide surveillance in a country of continental dimensions.

Nonetheless, the breadth and diversity of the dataset were sufficient to capture the main patterns of lineage turnover, reassortment, and interregional viral dissemination. A more pressing limitation, however, is the limited availability of standardized clinical and epidemiological metadata, which hinders efforts to link genomic data to disease severity, vaccine effectiveness, and patient outcomes [59].

In conclusion, this study provides a decade-long reconstruction of A/H1N1pdm09 evolution in Brazil, demonstrating that selective sweeps, potential reassortment events, and pandemic-related disruptions have shaped recent viral dynamics. By integrating genomic, spatial, and demographic data, our analyses highlight the value of Brazil’s strengthened post-COVID genomic surveillance network, which has enabled clearer detection of lineage replacement events and region-specific transmission patterns. Brazilian genomes also provide valuable temporal coverage from a large tropical – subtropical population, complementing datasets from temperate regions and enhancing the geographic breadth of global influenza surveillance. The prolonged circulation of certain lineages in Brazil further reveals transient viral diversity that may be underrepresented elsewhere, offering additional context for antigenic characterization and the early recognition of variants with potential phenotypic relevance.

In a global health landscape marked by recurring zoonotic spillovers and shifting public health priorities, scalable and real-time phylodynamic approaches, such as the one employed here, can play a key role in generating evidence to inform vaccine strain updates and strengthen pandemic preparedness. The persistent genetic diversification and ongoing circulation of A/H1N1pdm09 reinforce the need for sustained genomic surveillance and continued investment in public health infrastructure to monitor and mitigate the evolving burden of seasonal influenza effectively.

## Supplementary Material

Supplementary information.docx
