# Supplementary material for: Phylodynamic reconstruction of H1N1pdm09 influenza virus transmission in Brazil: a decade of evolutionary dynamics
Source: Emerg Microbes Infect. 2026 Jan 19;15(1):2620237. doi: 10.1080/22221751.2026.2620237 (PMC12857685; doi:10.1080/22221751.2026.2620237)
Supplement: Supplementary information.docx [file TEMI_A_2620237_SM5435.docx]

# Supplementary information


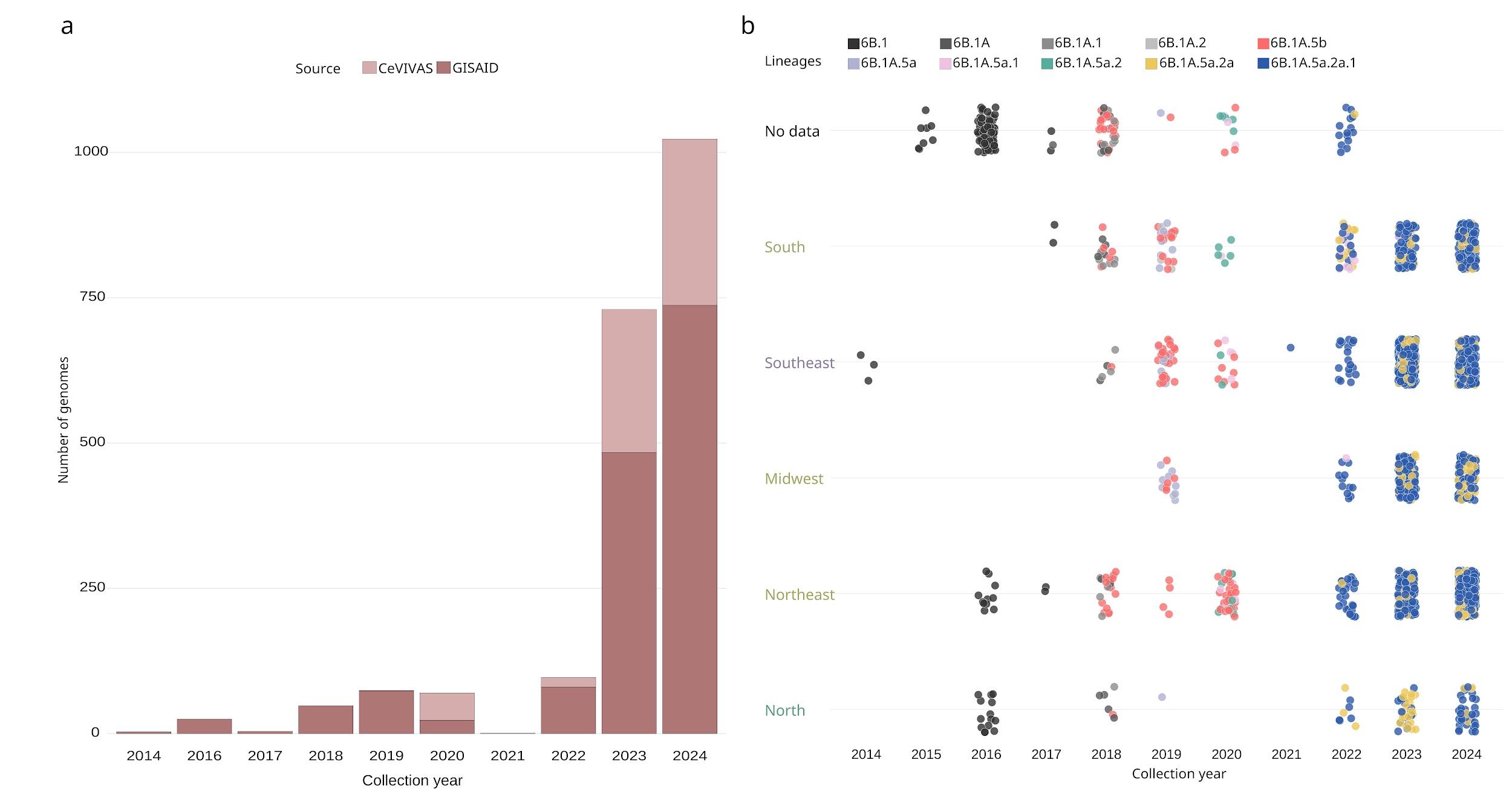


Figure S1. Dataset of H1N1pdm09 influenza A virus in Brazil. a) Annual distribution of the genomes analyzed in this study, classified by source: sequences newly generated by the CeVIVAS and those retrieved from GISAID. b) Spatial distribution of the genomes across Brazilian regions, with colors indicating distinct genetic clades. No data indicates a lack of information about the macroregion.


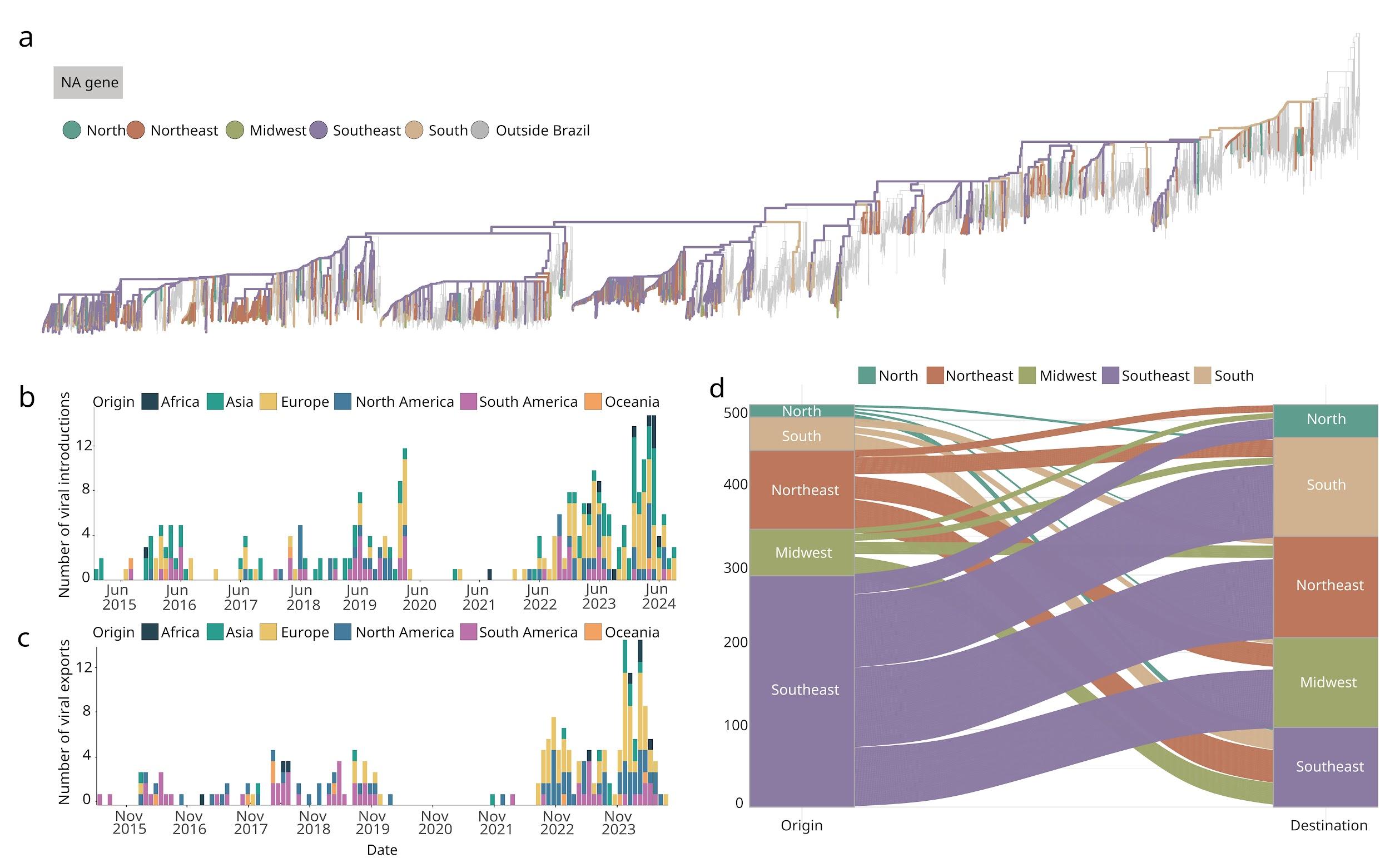


Figure S2. Phylogenetic and Evolutionary Dynamics of the NA gene of Influenza A/H1N1pdm09 in Brazil (2014–2024). a) Time-resolved maximum-likelihood phylogeny of NA gene, including high-quality complete genome sequences from Brazil (n = 597) generated in this study, analyzed alongside with other Brazilian and global reference sequences (n = 6,184); b) Number of viral introductions into Brazil, representing external entries from international sources; c) Number of viral exportations from Brazil to other regions of the world, representing internal sources of international spread; d) Number of intra-national viral exchanges between Brazilian macroregions, estimated by tracking state changes from the root to the tips of the phylogeny shown in panel a.


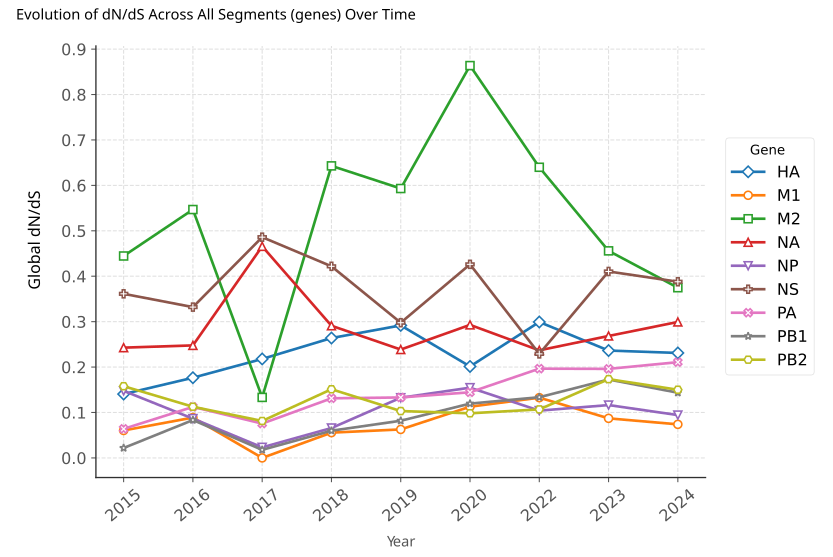


Figure S4. Selection pressures on Influenza A/H1N1pdm09 from Brazil over time (2015-2024). Yearly estimates of global dN/dS ratios for each gene segment were calculated using the SLAC method, with a significance threshold of p<0.05. Colored lines represent individual gene segments, including r: polymerase basic 2 (PB2), polymerase basic 1 (PB1), polymerase acidic (PA), haemagglutinin (HA), nucleoprotein (NP), neuraminidase (NA), two matrix proteins (M1 and M2), and non-structural proteins (NS). No data are available for 2021 due to the near-complete interruption of influenza genomic surveillance during the COVID-19 pandemic.


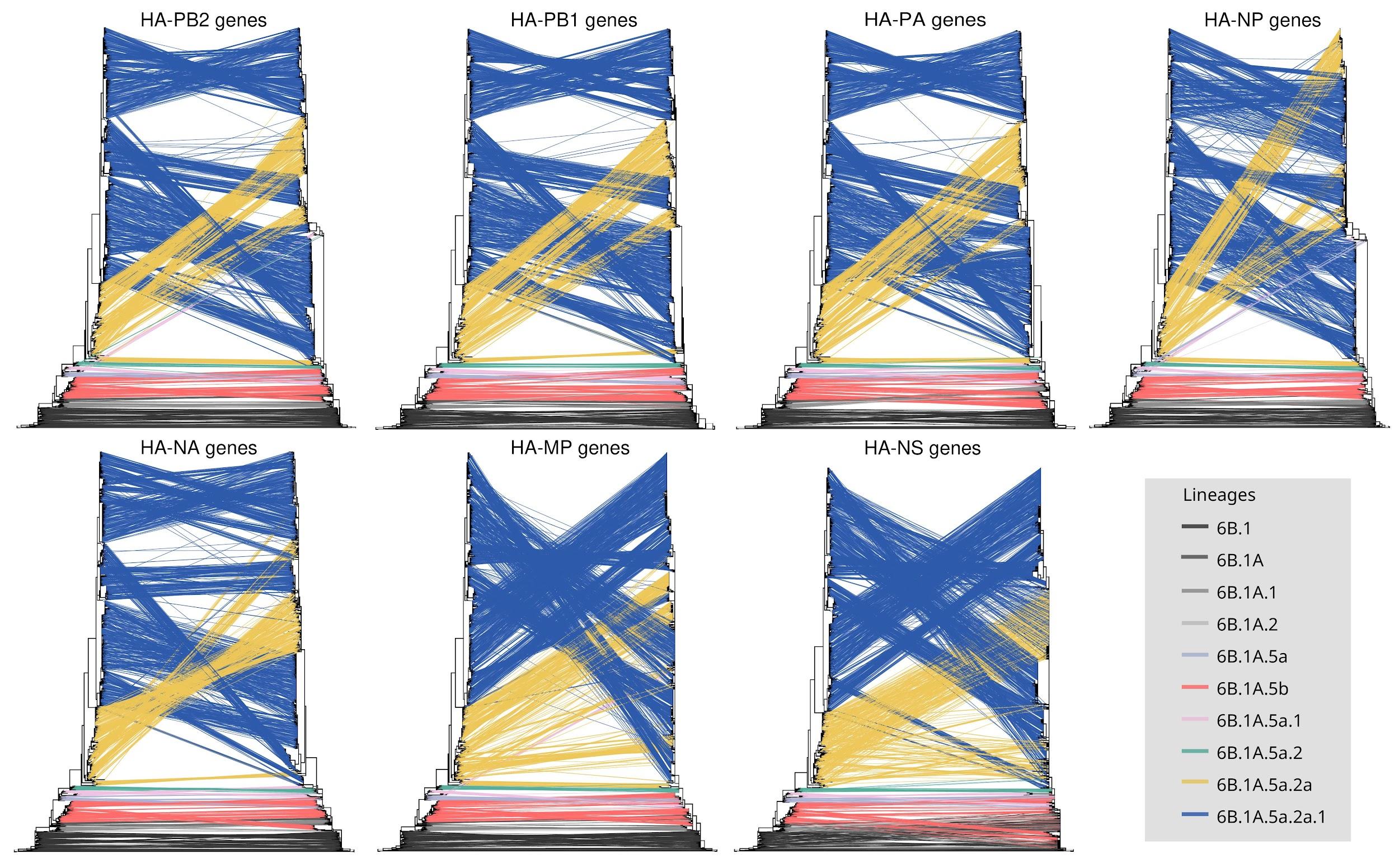


Figure S5. Phylogenetic incongruences among Influenza A/H1N1pdm09 gene segments in Brazil (2014–2024). Maximum-likelihood gene trees (HA vs. PB2, PB1, PA, MP, NP, NS) were reconstructed from aligned sequences, rooted to the oldest sampled genome. Tip labels were matched across trees, and taxa were color-coded by lineage. Normalized Robinson–Foulds (RF) distances between HA and other segments were: PB2 = 0.85, PB1 = 0.86, PA = 0.87, NP = 0.90, NA = 0.87, MP = 0.94, and NS = 0.94, indicating moderate to high topological divergence.


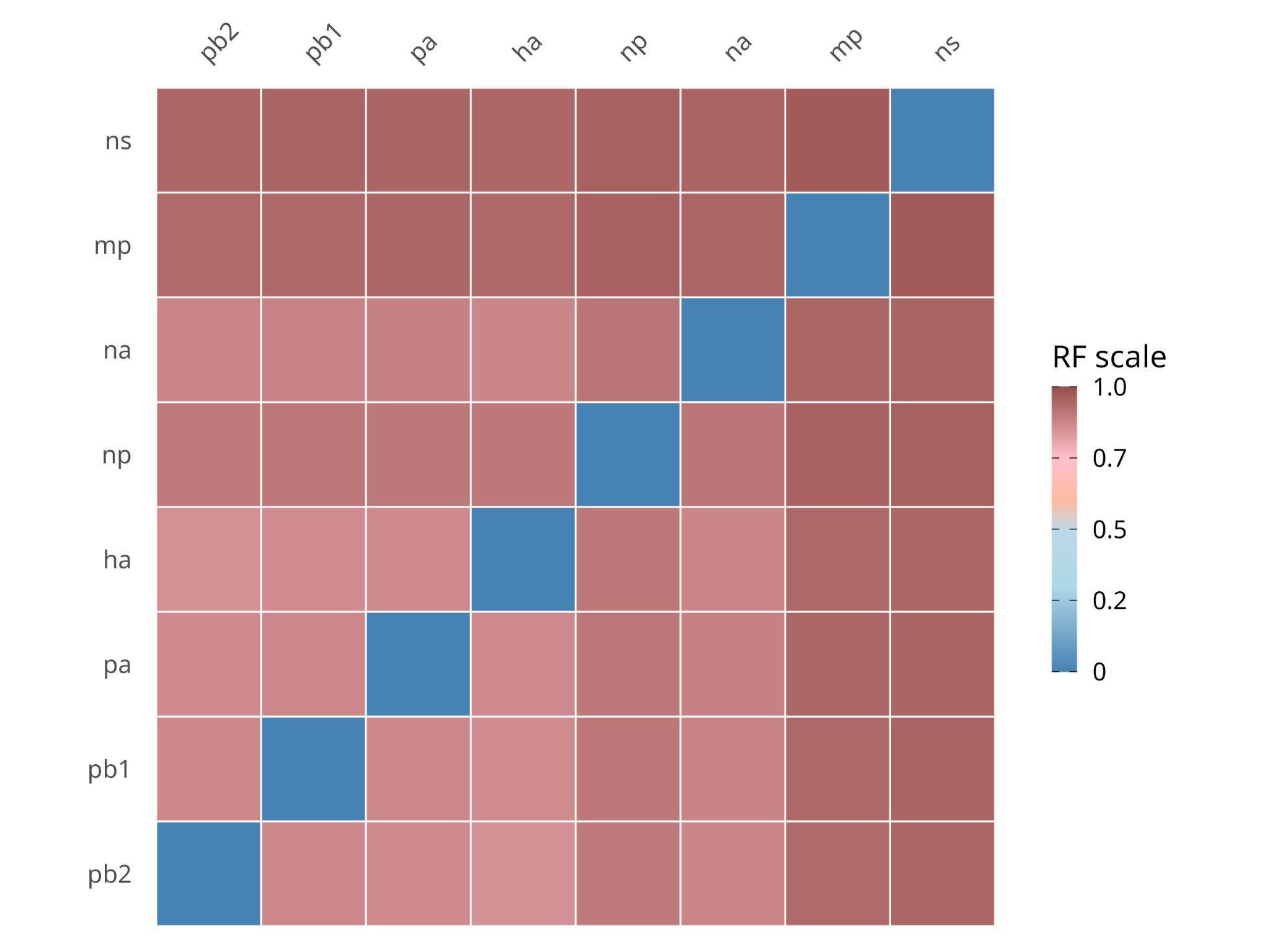


Figure S6. Heatmap of normalized Robinson–Foulds (RF) distances among maximum-likelihood phylogenetic trees inferred for the eight genomic segments of Influenza A/H1N1pdm09. The heatmap summarizes topological congruence among segment-specific trees, with RF values ranging from 0 (identical topology) to 1 (maximally different).


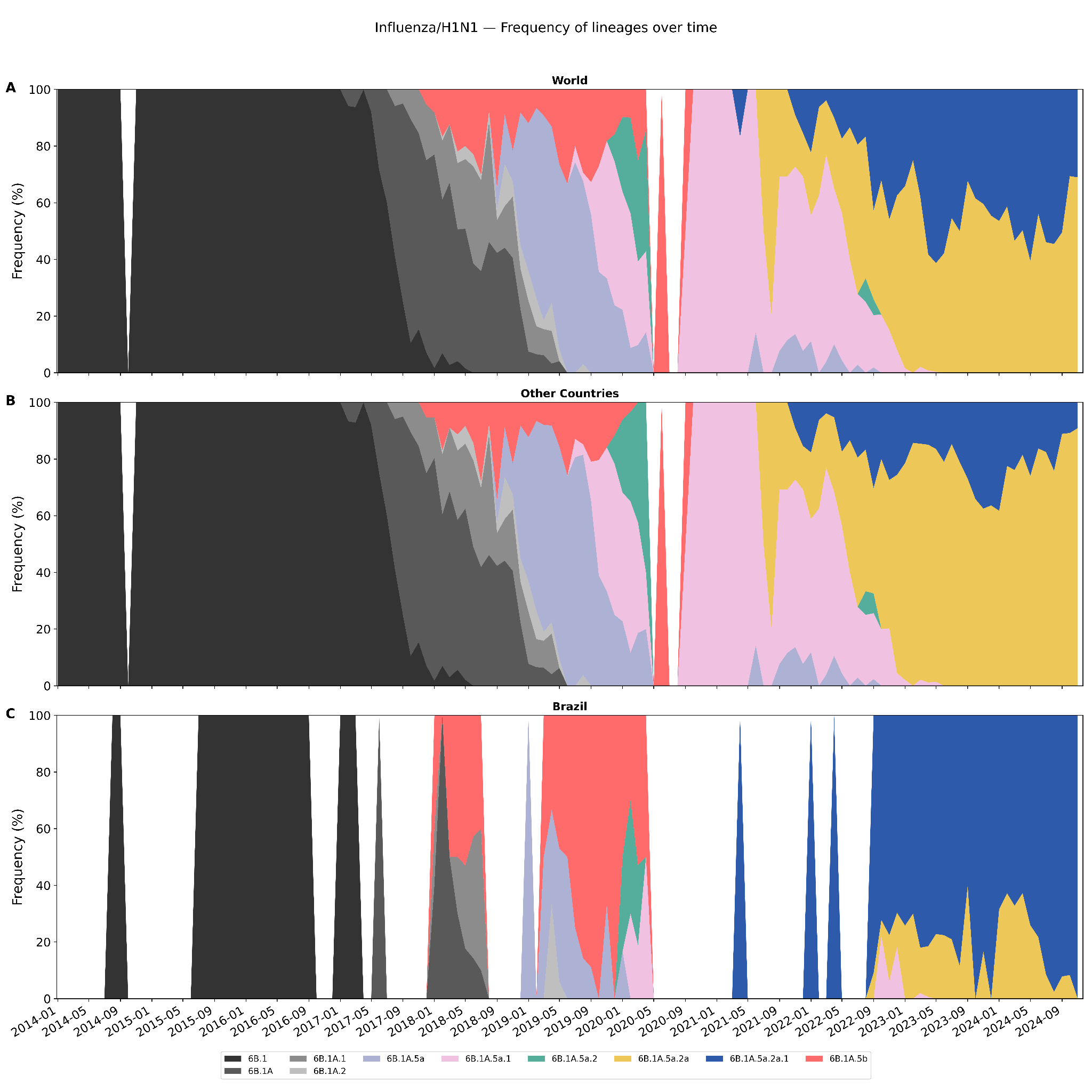


Figure S7. Frequency of A/H1N1pdm09 lineages over time. Panels display relative frequencies of A/H1N1pdm09 lineages from January 2014 to late 2024 for: a) global sequences. b) countries other than Brazilian sequences. c) Brazilian sequences. Colors represent lineages within influenza A/H1N1pdm09.

Table S3. Proportional representation of sequence data by continent and Brazilian samples.

| Year | Africa | Asia | Brazil | Europe | North America | Oceania | South America |
| --- | --- | --- | --- | --- | --- | --- | --- |
| 2014 | 73.53 | 0.00 | 8.82 | 14.71 | 2.94 | 0.00 | 0.00 |
| 2015 | 24.27 | 19.90 | 3.88 | 23.30 | 23.30 | 0.49 | 4.85 |
| 2016 | 16.30 | 19.46 | 23.60 | 10.71 | 10.71 | 2.43 | 16.79 |
| 2017 | 22.40 | 40.26 | 2.27 | 12.99 | 15.26 | 0.00 | 6.82 |
| 2018 | 14.55 | 20.97 | 12.52 | 14.55 | 17.37 | 0.78 | 19.25 |
| 2019 | 4.61 | 30.11 | 11.67 | 18.89 | 16.28 | 3.69 | 14.75 |
| 2020 | 14.98 | 17.42 | 28.22 | 16.72 | 13.24 | 45778 | 8.36 |
| 2021 | 65.52 | 15.52 | 0.86 | 10.34 | 7.76 | 0.00 | 0.00 |
| 2022 | 18.72 | 23.10 | 19.06 | 20.40 | 10.79 | 1.52 | 6.41 |
| 2023 | 08.03 | 18.83 | 43.09 | 13.75 | 10.80 | 0.18 | 5.31 |
| 2024 | 5.48 | 15.04 | 55.54 | 13.19 | 6.84 | 0.60 | 3.31 |

Table S4. Demographic information of A/H1N1pdm09 sequences generated in this study, collected in Brazil between 2020 and 2024.

| Age Group | Female (n) | Male (n) | Total (n) | Female (%) | Male (%) | Total (%) |
| --- | --- | --- | --- | --- | --- | --- |
| <2 | 10 | 12 | 22 | 45.5 | 54.5 | 3.7 |
| 2–10 | 31 | 36 | 67 | 46.3 | 53.7 | 11.2 |
| 11–20 | 33 | 33 | 66 | 50.0 | 50.0 | 11.1 |
| 21–35 | 67 | 57 | 124 | 54.0 | 46.0 | 20.8 |
| 36–50 | 86 | 63 | 149 | 57.7 | 42.3 | 25.0 |
| 51–65 | 57 | 45 | 102 | 55.9 | 44.1 | 17.1 |
| 66–80 | 22 | 21 | 43 | 51.2 | 48.8 | 7.2 |
| >81 | 14 | 10 | 24 | 58.3 | 41.7 | 4.0 |

Table S5. Positively selected sites inferred from global non-synonymous to synonymous substitution rate ratios (dN/dS) using Single-Likelihood Ancestor Counting (SLAC) and Fast, Unconstrained Bayesian AppRoximation (FUBAR) methods implemented in HyPhy.

| Segment (gene) | Global dN/dS (SLAC) | Sites (SLAC) | Global dN/dS  (FUBAR) | Sites  (FUBAR) | Shared Sites |
| --- | --- | --- | --- | --- | --- |
| HA | 0.2379 | 6, 11, 62, 86, 158, 233 | 0.1776 | 6, 62, 86, 154, 158, 202, 233, 277 | 6, 62, 86, 158, 233 |
| M1 | 0.0824 | - | 0.0760 | - | - |
| M2 | 0.4905 | 21, 23 | 0.6405 | 10, 21, 23, 82 | 21, 23 |
| NA | 0.2857 | 77, 321 | 0.2028 | 77, 80 | 77 |
| NP | 0.1046 | 186, 217, 313 | 0.1016 | 34, 186, 217, 257, 313 | 186, 217, 313 |
| NS | 0.3847 | 84, 178, 205 | 0.5819 | 81, 84, 101, 178, 205, 213 | 84, 178, 205 |
| PA | 0.1869 | - | 0.1524 | 343 | - |
| PB1 | 0.1408 | 384, 460 | 0.1268 | 384, 460 | 384, 460 |
| PB2 | 0.1487 | 184 | 0.1268 | 184, 194, 451 | 184 |
